# A community-based group-guided self-help intervention for low mood and stress: study protocol for a randomized controlled trial

**DOI:** 10.1186/1745-6215-14-392

**Published:** 2013-11-19

**Authors:** Carrie-Anne McClay, Jill Morrison, Alex McConnachie, Christopher Williams

**Affiliations:** 1 Institute of Health and Wellbeing, University of Glasgow, Gartnavel Royal Hospital, Administration Building, 1055 Great Western Road Glasgow, Glasgow City G12 0XH, UK; 2 General Practice and Primary Care, Institute of Health and Wellbeing, University of Glasgow, 1 Horselethill Road, Glasgow G12 9LX, UK; 3 Robertson Centre for Biostatistics, Boyd Orr Building, University of Glasgow, Glasgow G12 8QQ, UK

**Keywords:** Depression, CBT, Guided self-help, Classes, Groups, Psychotherapy, RCT, Voluntary sector, Treatment gap, Economic analysis, Bibliotherapy, Living Life to the Full, LLTTF, Low mood, Distress, Anxiety

## Abstract

**Background:**

Depression is a mental health condition which affects millions of people each year, with worldwide rates increasing. Cognitive behavioral therapy (CBT) is recommended in the National Institute for Health and Clinical Excellence (NICE) guidelines for the treatment of depression. However, waiting lists can cause delays for face-to-face therapy. Also a proportion of people decline to present for help through the health service – the so-called treatment gap. Self-referral to CBT using community-based group interventions delivered by a voluntary sector organization may serve to resolve this problem. The aim of this randomized controlled trial (RCT) is to determine the efficacy of such a guided CBT self-help course, the ‘Living Life to the Full’ (LLTTF) classes delivered by the charity Action on Depression (AOD). The primary outcome is level of depression at 6 months assessed using the patient health questionnaire-9 (PHQ9) depression scale. Secondary measures include levels of anxiety and social functioning.

**Methods/design:**

Participants with symptoms of low mood will be recruited from the community through newspaper adverts and also via the AOD website. Participants will receive either immediate or delayed access to guided CBT self-help classes - the eight session LLTTF course. The primary endpoint will be at 6 months at which point the delayed group will be offered the intervention. Levels of depression, anxiety and social functioning will be assessed and an economic analysis will be carried out.

**Discussion:**

This RCT will test whether the LLTTF intervention is effective and/or cost-effective. If the LLTTF community-based classes are found to be cost effective, they may be helpful as both an intervention for those already seeking care in the health service, as well as those seeking help outside that setting, widening access to psychological therapy.

**Trial registration:**

Current Controlled Trials ISRCTN86292664

## Background

Mood disorders were the most prevalent psychiatric hospital discharge diagnosis in women in Scotland in 2012 [1]. In 2011/12 the total cost of antidepressant medication prescribed in Scotland was £31.4 million [2]. There is evidence for the role of social and environmental influences on the development and course of depressive disorders [3]. The World Health Organization states that, in the majority of cases, depression can be effectively treated within primary care [4]. However, fewer than 50% of patients receive the necessary medication or psychotherapeutic treatments [5]. This treatment gap may have occurred for a number of reasons, including non-recognition of the problem, non-presentation as a result of a fear of stigma, or limited knowledge about treatment options.

Community surveys have confirmed that members of the public give higher endorsements to self-help, voluntary sector and local support networks than for more formal treatment approaches [6]. Interventions that emphasize community-based recruitment, self-referral and delivery of treatment courses via a voluntary sector group may engage people who would otherwise not receive appropriate help for depression within the health service, and may reduce the burden on the health service by providing an alternative treatment path for those who are already engaged. Voluntary sector groups are popular and provide important peer support. However, there may be advantages of adding in an evidence-based structure and content to the group sessions to enhance outcomes from this delivery approach.

### Cognitive behavioral therapy

The National Institute for Health and Clinical Excellence (NICE) recommends cognitive behavioral therapy (CBT) for mild-to-moderate depression [7]. Traditional CBT consists of 12 to 20 1-hour sessions with a mental health expert and can be delivered in primary care settings [8]. However, it remains difficult to provide this *high-intensity* (HI) specialist CBT due to the large volume of patients with low mood, and as a result waiting lists are long. An alternative is to supplement HI delivery with *low-intensity* (LI) CBT [9]. Several separate strands of LI delivery exist, including CBT delivered using bibliotherapy (written self-help books) and computerized CBT (cCBT) [10], as well as LI forms of classes/groups and behavioral activation. These are recommended as part of stepped-care approaches before HI CBT for mild-to-moderate depression. NICE [7] and Gellatly and colleagues [11] recommend that including guidance/support in using bibliotherapy and cCBT significantly improves outcomes for those with depression using these approaches. Crucially, the support does not need to be delivered by a mental health or CBT expert and the focus can be on supportive monitoring [11].

Such support is often provided on an individual basis by workers such as self-help coaches or psychological wellbeing practitioners. Self-help strategies with support may be as effective as HI specialist CBT for some [12], and are also a preferred treatment option for many users [6]. Voluntary sector support is also viewed as attractive and avoids a formal mental health label and diagnosis. However, LI working usually relies on one-to-one support either face-to-face or by phone, and is still relatively time consuming.

An alternative is to provide access to specialist delivered face-to-face group therapy led by expert mental health practitioners. Existing HI specialist group CBT approaches include behavioral therapy groups consisting of 12 sessions, initially held twice weekly [13] attended by 6 to 10 adults and led by two highly trained group leaders. Such approaches are helpful [7] but are very therapist-intensive and access for patients is restricted.

Currently there are few face-to-face LI classes available and none have been adequately tested in a randomized controlled trial (RCT) setting. White has tested large psycho-educational classes for anxiety [14]; however, these do not specifically target depression. Brown and colleagues [15] reported the results of a large group class based on a widely used CBT book about confidence. Their classes included a high proportion of people with depression and focused on teaching how to apply a written self-help resource (bibliotherapy) for self-confidence, ‘*Overcoming Low Self-esteem’*[16]. They were, however, delivered in a single one-off class with no on-going guidance or support for the application of the CBT self-help resources. NICE [7] identifies no LI guided CBT classes and no studies of LI-CBT classes that address depression and include health economic outcomes. It recommends classes with smaller numbers of attendees, rather than large group classes, and separately recommends the use of guided CBT self-help resources for guided/supported use by individuals.

This will be the first study to bring together these different strands by evaluating CBT self-help resources specifically for depression delivered with LI support/guidance via short, weekly, face-to-face, small group classes delivered through the voluntary sector. Our project draws on current NICE guidelines and offers the possibility of rapid access and larger throughput of numbers coupled with delivery by non-specialist trainers. The classes will be delivered by voluntary sector workers with a non-National Health Service (NHS) label (the mental health charity Action on Depression (AOD) - formerly Depression Alliance Scotland) in order to appeal to a wide range of people including those currently attending the NHS as well as those who are not. They will also allow self-referral, an approach which has been successfully used previously by Brown and colleagues [17] and which found that approximately one-third of the sample had mild depression, 37% had moderate/severe depression and 18% were in the ‘extremely severe depression’ category according to baseline Beck Depression Inventory [18] scores.

### The Living Life to the Full classes

The Living Life to the Full (LLTTF) classes contain eight life skills sessions that teach a range of CBT-based life skills in a classroom setting based in a locally accessible location such as a library or hotel. Classes are attended by up to 16 people and last 90 minutes. Each weekly class focuses on a different common problem faced by people when they feel low or anxious. The class content is: *1) Why do I feel so bad? 2) I can’t be bothered doing anything. 3) Why does everything always go wrong? 4) I’m not good enough (low confidence). 5) How to fix almost everything. 6) The things you do that mess you up. 7) Are you strong enough to keep your temper? 8) Ten things you can do to help you feel happier straight away.* The classes use everyday terms and avoid professional terminology. They are designed to encourage an individualized plan to be made at the end of each session using a Plan, Do, Review structure [19]. Each class consists of slides, course leader notes/scripts, worksheets and a linked booklet that summarizes the topic and includes work tasks to facilitate practice at home. The classes offer guided CBT for depression delivered over a series of eight class-based sessions delivered over eight weeks. Each class is delivered using a mix of didactic teaching, question and answer discussion and tasks in groups or pairs. There are currently no equivalent classes using the same model of delivery. This accessibility is reinforced by the language used (that is, the “class” not “group”, “life skills training” instead of “CBT” and “low mood and stress” rather than “depression and anxiety”). The way of communicating CBT is highly accessible [20] and effective [21,22]. The language used in the classes includes everyday terms to describe symptoms such as stress, distress and low mood rather than the more formal diagnostic terms depressive disorder, depression and anxiety, and so on. The aim is to be inclusive and appealing to participants who are being recruited through a community-based initiative.

A ninth session, Planning for the Future and Reunion, is held 6 weeks after the final class. The class takes place about 6 weeks after the core 8-week course as it is thought that this is an appropriate duration for participants to integrate/apply the skills they have learned in the course in their everyday lives. The revision class therefore serves as a booster session to keep participants on track with the intervention, as they are advised to continue using the resources after the group support ends for optimal benefit.

### Results of pilot studies

The classes are used in Scotland as part of the Widening Access to Self-help project an extension of the Scottish Government funded Doing Well by People with depression program [23] and have proved popular and accessible. An independent Health and Social Board national pilot study conducted by AWARE Defeat Depression, a charity in Northern Ireland, evaluated 46 groups (356 individuals) who took part in the classes. The General Health Questionnaire-12 [24] found only 9.8% of participants self-rated as ‘Happy’ at the start of the intervention compared to over 60% at the end, which was a statistically significant change (*P* < 0.001). Additionally, 77% rated themselves as being in the ‘Depressed’ category before the program, with only 21% depressed post-intervention. The classes also significantly improved knowledge and understanding in relation to stress and low mood. In Scotland, we have recently run a pilot of the face-to-face classes also with statistically significant improvements in low mood. Also, the Equally Connected Equality Team at NHS Lothian ran the LLTTF classes as a pilot in Black and Minority Ethnic settings. They found that 100% of respondents agreed or strongly agreed that the classes had been helpful [25].

However, to date there have been no large RCTs of the classes and NICE has identified a need for such studies with a health economic component to be completed. We have, therefore, recently undertaken a pilot RCT using the same design as the current application. This has allowed us to test and be confident of our recruitment process, establish power and test the delivery of the intervention and research. In a previous pilot in Glasgow and Northern Ireland (with course delivery by the charities AOD and AWARE), we successfully screened and randomized 53 participants using the same planned recruitment strategy. Mean differences at the primary follow-up point (3 months) have informed our power calculation (see below).

We have therefore successfully tested recruitment, questionnaire delivery and data collection, randomization and delivery of the face-to-face classes in the community. This substantive RCT differs in so far as it has been modified to include a 6 month rather than 12 week primary endpoint and is powered to definitively answer the question of whether the LLTTF course is an effective treatment for low mood. The 6 month primary follow-up point will allow a longer-term impact of the intervention to be assessed and groups can be compared at this time point whilst the delayed access control (DAC) remain a control group. As this is a long-term follow-up point, participants may seek additional support prior to follow-up; data on services accessed during the study will be collected at 6 months using the Client Service Receipt Inventory (CSRI).

### Aims

This is an RCT comparing class-delivered guided CBT self-help using the LLTTF classes, with a DAC who will receive the intervention after 6 months. We will follow-up participants until 6 months, which represents a reasonable level of sustainability. Our trial experience and recent review makes us feel that the delayed treatment arm as opposed to treatment as usual is necessary to maximize recruitment and retention for this population [26]. To address stigma and encourage self-referral (known to address the treatment gap), both recruitment and delivery will take place in community settings and recruit directly through community-based adverts supplemented with advertisements through AOD.

The aim of this substantive study is to recruit people experiencing significant depression, and to include both those already receiving NHS support as well as those who are not. We will include both those with clinically diagnosed major depression as well as those with significantly raised mood scores. We will describe the population in detail in terms of mood severity and clinical diagnosis. The study will assess the effectiveness of LLTTF in reducing symptoms of depression and anxiety and in improving social functioning. Effectiveness will be measured using standardized outcome measures that address a broad range of outcomes. The cost effectiveness of this intervention will also be investigated using the CSRI [27] to estimate personal and healthcare costs and the EuroQuol EQ5D [28] as a measure of quality of life.

### Research questions

#### Primary question

Do the LLTTF classes result in an improvement in symptoms of depression and anxiety at 6 months compared to a DAC group, as measured by the patient health questionnaire-9 (PHQ9) [29] and generalized anxiety disorder 7 (GAD7) [30].

#### Secondary questions

a) Do the LLTTF classes result in an improvement in symptoms of depression and anxiety at 6 months compared to DAC, as measured by the PHQ9 and GAD7 for those with a baseline PHQ9 score of 10+ and those with a score of 5 to 9?

b) Do the LLTTF classes result in an improvement in social function at 6 months compared to DAC as measured by the Work and Social Adjustment Scale (WSAS) [31]?

c) Are the LLTTF classes cost effective?

d) Are the LLTTF classes satisfactory to participants?

## Methods/design

### Overview

**Figure 1:**
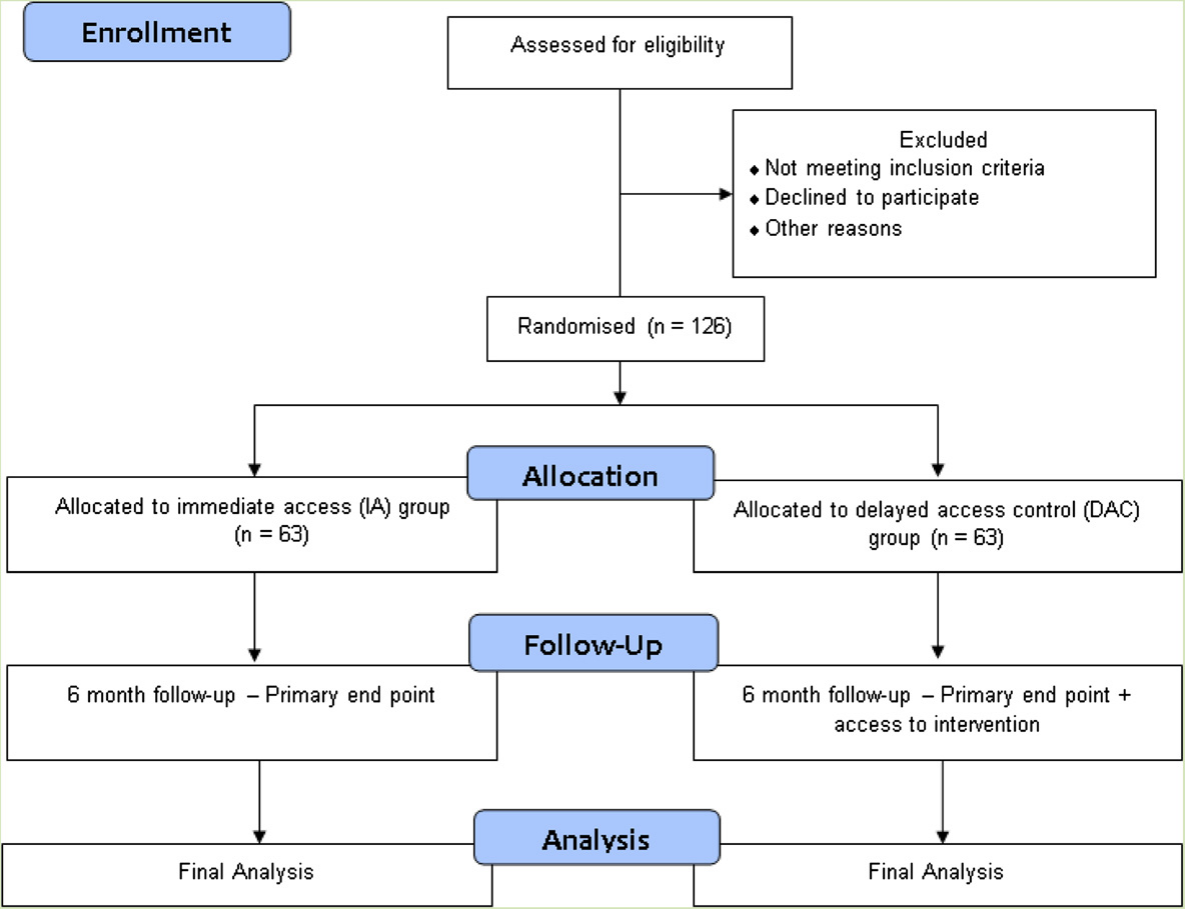
Flow diagram of the randomized controlled trial.

This is a pre-post design RCT with DAC and the primary outcome at 6 months follow-up (see flow diagram in Figure 1). It explores the efficacy, take up and acceptance of CBT-based community delivered classes for adults with low mood. The study does not recruit from the health service and has been granted ethical approval by the College of Medical, Veterinary & Life Sciences Ethics Committee for Non Clinical Research Involving Human Subjects, University of Glasgow, Ref. 2012065.

#### Participants

Recruitment of people with depressive symptoms (defined below and including both those already separately seeking NHS help, and those who are not) will be carried out using multiple community-based methods including: posters, Google advertisements, Metro newspaper advertisements and via AOD websites, phone support line, newsletters and local groups. No participants will be recruited via the NHS and we will recruit exclusively from Scotland.

#### Inclusion criteria

Individuals aged 16 or over with at least mild depressive symptoms defined as a score of 5 or more on the PHQ9.

#### Exclusion criteria

Participants are excluded if they are aged under 16, they cannot read, speak and understand English, they cannot travel to the classes, they do not consent to abide by normal social etiquette within the classes, their score on the PHQ9 is below 5 (reflecting minimal symptoms) or they are currently receiving psychotherapy/talking therapy or counseling. We will not exclude for comorbidity, severity of depression or risk; however, participants throughout will be provided with contact details and instructions on what to do if they feel worse.

### Setting and procedure

Individuals will find out about the study through reading adverts in the Metro free-sheet (Scottish edition), or seeing an online or other contact from AOD which will direct them to contact the Research assistant by phone or email for further details. Anyone who expresses interest in the study will be sent copies of the Participant Information Sheet (which includes information on how to seek help if it is needed immediately), brief Eligibility Questionnaire, PHQ9, GAD7, Hospital Anxiety and Depression scale (HADS) [32] and Consent Form.

The eligibility questionnaire collects basic demographic information including age, gender, information regarding previous or ongoing mental health treatment (including current psychotherapy, antidepressant medication and/or counseling), ability to travel and attend classes (including location and time preferences) and chronicity of their low mood (self-reported in months/years). The questionnaires will be completed and returned by post with the consent form allowing eligibility to be assessed.

Eligible participants will then be invited to take part in a telephone call to confirm their diagnosis using the Mini International Neuropsychiatric Interview (MINI) [33]. We anticipate that most, if not all, participants will agree to the interview. However, for some people an attraction of the voluntary sector delivery is that they can seek help without creating a formal NHS record. Some potential participants may therefore also rather not take part in a diagnostic interview, so individuals who enter the study will be given the option to decline the interview if they prefer. The MINI will be useful in describing the population being studied but will not be used as an outcome measure. When sufficient numbers have been recruited to fill two classes, the baseline questionnaires will be sent for all participants to complete and participants will be informed of their group allocation.

#### Randomization

Randomization will be carried out at the Robertson Centre for Biostatistics at the University of Glasgow, part of the Glasgow Clinical Trials Unit, by staff not in direct contact with participants, and who will not carry out the final analysis. Randomization will occur each time sufficient participants have been recruited to fill two classes (up to 16 participants per class). Overall, we anticipate needing to run five immediate access (IA) classes and 5 DAC classes. Randomization will take into account timing of classes (availability to attend evening or daytime classes), location of classes (that is, classes in either Edinburgh or Glasgow), and severity of depression as measured by the PHQ9 (score of less than or equal to 9 and greater than or equal to 10) at the eligibility stage. Participants will be assigned to either IA or to DAC. Participants will then be given the date, time and place (IA or DAC) where their allocated classes will be held.

#### Immediate access group

Participants randomized to IA will be informed that they will begin the LLTTF classes within 2 to 3 weeks.

#### Delayed access control group

Participants randomly allocated to the DAC group will be informed that they will begin attendance at the classes after a delay of 6 months. All participants will continue with any usual treatment as required. Use of anti-depressant medication and any new or ongoing mental health support will be recorded at follow-up using the CSRI.

#### Intervention: the LLTTF classes

LLTTF class sessions last 1.5 hours including refreshments and cover the topic areas described earlier. Sessions are presented usually in library rooms using a consistent slide set and presenter support scripts which have accompanying linked booklets and worksheets. Two class leaders co-present and aim to cover the core content in both a friendly and engaging manner. Adherence to the delivery method will be assessed by a research assistant sitting in on a selection of classes and rating the delivery of key points and delivery quality to provide an overall acceptable or unacceptable quality rating. The checks cover two components: firstly, adherence to the content (core concepts are covered); and, secondly, the presentation skills of the presenter, both recorded on a scoring checklist.

### Measures

The following measures are used in the study (see Table 1).

**Table 1:** Timing of measures

**Screening**	**Baseline**	**6 months (IA)**	**6 months (DAC)**
PHQ 9	PHQ 9	PHQ 9	PHQ 9
GAD7	GAD7	GAD7	GAD7
HADS	HADS	HADS	HADS
Demographics including presence of “hard to engage” groups (for example, young men) MINI diagnostic interview	WSAS	WSAS	WSAS
	CSRI (6 months retrospective)	CSRI	CSRI
		EQ5D	EQ5D
		CSQ-8	CSQ-8
		“Your experience of attending the ‘Living Life to the Full’ class” Questionnaire	

CSQ-8, an 8-item client satisfaction questionnaire; CSRI, Client Service Receipt Inventory; DAC, delayed access control; GAD7, generalized anxiety disorder 7; HADS, Hospital Anxiety and Depression scale; IA, immediate access; MINI, Mini International Neuropsychiatric Interview; PHQ9, patient health questionnaire-9; WSAS, Work and Social Adjustment Scale; EQ5D, the 5 item EuroQuol questionnaire of quality of life.

### Mini international psychiatric interview, version 6.0

The MINI is a widely used psychiatric measure [33]. The ‘Major depressive episode’ module of this interview will be completed with participants who provide additional consent for this assessment. Interviewees are asked to respond ‘yes’ or ‘no’ to a series of questions concerning their mood both in the last 2 weeks and during their most symptomatic past episode (also a 2-week period). For some items, the interviewer asks for examples to clarify responses. Participants are considered as having a current episode of depression if they have felt depressed or down most of the day nearly every day for the past 2 weeks, if they have had a significant loss of interest in usually enjoyable activities in the last 2 weeks and if they respond ‘yes’ to a total of five or more of the depressive symptoms they are asked about. Past episodes of low mood are scored in the same way. Validation studies of the MINI have demonstrated that it is an efficient, reliable and valid measure that can be successfully used to make DSM-IV and ICD-10 diagnoses [33].

### Patient health questionnaire-9

The primary outcome (PHQ9 depression scale) is at 6 months using an intention-to-treat analysis. PHQ9 is a freely available mood rating questionnaire consisting of nine questions mirroring DSM-IV depression diagnostic criteria [29]. The PHQ9 has shown diagnostic validity in a study of 3,000 adult patients. In this study, PHQ9 diagnosis rates were comparable with diagnosis by a mental health professional [29]. Each item is rated on a scale of 0 to 3, giving a maximum score of 27. Cut-off scores are used to label depression severity as: 0 to 4, minimal depression; 5 to 9, mild depression; 10 to 14, moderate depression; 15 to 19, moderately severe depression; 20 to 27, severe depression.

### Generalized anxiety disorder 7

The GAD7 is a seven-item questionnaire focusing on symptoms of anxiety experienced in the past 2 weeks [30]. Each item is rated according to the frequency of the described problem. The responses are scored as follows: 0 = ‘not at all’, 1 = ‘several days’, 2 = ‘more than half the days’, 3 = nearly every day’. Therefore the maximum score is 21. Studies of this measure have resulted in the authors stating the following cut of scores: 5 to 9, mild anxiety; 10 to 14, moderate anxiety; and 15 and above, severe anxiety. The GAD7 showed good reliability and criterion, construct, factorial, and procedural validity in a study carried out by the authors in 15 primary care clinics [30].

### Hospital anxiety and depression scale

We will also use the HADS as a secondary check of depression and anxiety as it is widely used in similar research projects [32]. The HADS is a 14-item questionnaire with seven of these items assessing level of anxiety and seven assessing depressive symptoms. Each item is rated on a scale from 0 to 3; therefore, there is a maximum score of 21 for anxiety and 21 for depression. The higher the score, the more severe are the anxious or depressive symptoms. The HADS was found to be a reliable measure of anxiety and depression in a large study of out-patients. Furthermore, the subscales are valid, with higher scores indicating greater severity of the disorder [32].

### Work and social adjustment scale

The WSAS will be used to assess levels of social functioning [34]. The WSAS is a five-point questionnaire addressing life activities. The questionnaire probes issues relating to the impact the disorder in question is having on the individual’s everyday life and functioning. Responses are given on a scale of 0 to 8, with higher scores indicating higher level of impairment or disruption to social functioning. Therefore, the maximum score on this questionnaire is 40. Mundt and colleagues [34] state that, in light of studies that included two clinical groups, in those with depression and those with obsessive-compulsive disorder, a score below 10 on the WSAS does not indicate clinically significant problems and between 10 and 20 indicates significant impairment. Those scoring over 20 are likely to have significant problems in their social functioning [34]. The WSAS has strong psychometric properties, showing good reliability and validity in patients with depression and in a sample of patients with obsessive-compulsive disorder, with greater severity of symptoms being strongly associated with functional impairment [34].

### The client satisfaction questionnaire

The client satisfaction questionnaire (CSQ-8) will be administered post-intervention as a measure of satisfaction with the intervention. The CSQ-8 is an eight-item questionnaire rated using a four-point Likert scale. Scores range from 8 to 32 with higher scores indicating greater satisfaction with the intervention in question. A series of studies of this measure have indicated high internal consistency (Cronbach’s alpha 0.92 to 0.93 for eight-item scale) as well as criterion and construct validity [35].

### Follow-up

Participants will be offered a £10 Amazon/Tesco voucher in return for completed follow-up questionnaires. This is expected to improve follow-up rates and help us to achieve our target of getting at least a 70% response rate. Additionally, in order to maintain contact with participants during the delay before follow-up assessments are posted out, greetings cards or letters will be sent. In these, the IA participants will be given details of their final Planning for the Future/Revision class and next assessment point. The DAC participants will be thanked for their patience, given details of the next evaluation assessment and will be given an approximate start date for their course.

#### Statistical power and sample size

The primary analysis will compare changes in PHQ9 scores at 6 months between intervention groups in all participants. We have powered the study, however, to be able to detect a between-group difference of 5.5 points on the PHQ9 score within the more severe subgroup of participants scoring 10 points or more on the PHQ9 at baseline. A difference of 5.5 points is used to reflect a category change on the PHQ9 and is clinically significant as well as statistically significant. Preliminary pilot RCT data, from a mixture of intervention and control group participants, showed that for those with a PHQ9 score of 10 or more at baseline, mean PHQ9 scores reduced from 17.7 to 10.8 points after the intervention, with a standard deviation of the changes of 6.1 points.

Based on a two-sample *t*-test, a sample size of 27 participants per arm would be required to have 90% power. In the pilot, follow-up data at the primary endpoint were available for approximately 65% of those randomized. To allow for this, 84 people (two groups of 42) with PHQ9 scores of 10 or more would need to be randomized. In the pilot, one-third of participants had PHQ9 scores less than 10 points at baseline, so that 126 participants in total (two groups of 63) will be randomized in this trial. We will ensure that the required number of participants scoring 10+ on the PHQ9 have entered the study before ending recruitment.

Our pilot study strongly supports our potential to recruit 126 participants using our multi-point recruitment strategy. We have not accounted for any clustering effects in these calculations, since clustering can only occur within the intervention group (control participants will not meet in a group setting during the first 6 months of the study), and the estimate of variation in the primary outcome used in the above calculation is conservative in that it includes a component due to clustering within intervention groups. As part of the analysis we will, however, assess the extent to which the study findings are sensitive to any clustering effects.

#### Statistical analyses

The primary analysis will use analysis of covariance, testing the difference between groups in PHQ9 and GAD7 scores in all participants at 6 months with adjustment for baseline values. This is more efficient than two-sample *t*-tests, increasing the power of the study. An intention-to-treat approach to analysis will be used.

A criticism sometimes made of research studies that allow self-referral and utilize community-based recruitment is that patients are not significantly depressed. Planned secondary analyses will therefore be carried out for those with PHQ9 scores of 10 or more (that is, moderately severe depression), and for those with scores less than 10, at baseline. We will test whether the intervention effect interacts with baseline PHQ9 score (in other words, is the intervention more or less effective dependent upon the baseline PHQ9 score). The model for all participants will also be extended to assess the impact of baseline participant characteristics including age, gender, antidepressant use, level of depression and measures of compliance with the intervention (and, for those factors that influence outcome, their interaction with intervention). Similarly, regression methods, chosen according to the distribution of outcome variable being investigated, will be used for other outcomes. Simple and multiple imputation methods will be explored for the primary outcome to assess the sensitivity of the main study findings to alternative assumptions regarding missing data.

#### Economic analysis

The CSRI and EQ5D will be used to measure use of services and current state of health; these will be used in the health economic analysis of the intervention. Economic analysis will take a health service perspective as recommended by NICE. We shall apply generalized linear models for costs and health utilities, with bootstrapping and the method of recycled predictions to estimate the mean and uncertainty in the Incremental Cost Effectiveness Ratio [36]. Interpretation will be aided using cost-effectiveness acceptability curves derived using the net-benefit approach with values between £0 and £100,000 placed on a quality-adjusted life year gain so as to include the threshold used by NICE.

We shall assess the cost-effectiveness of the LLTTF classes in relation to baseline participant characteristics, including PHQ9 score, age, gender and antidepressant use. We shall assess the sensitivity of our findings to variations in the cost of delivering the intervention.

## Discussion

### Importance of the project

If this RCT demonstrates that the intervention is effective and cost effective then this has important implications for healthcare delivery. We hope that the community recruitment and AOD support will include people into the project who might otherwise fail to engage in traditional NHS-based services thus addressing the wider treatment gap. The design allows a detailed description of those recruited in order to understand the current treatment, severity, chronicity, diagnostic category and impact of those recruited.

As well as testing the effectiveness and cost-effectiveness of the intervention, the design also tests the attractiveness of partnership delivery through the voluntary sector - an area where outcome data are often poorly available.

### Benefits to participants

Participation within this research will allow adults who are experiencing symptoms of low mood/depression to take part in CBT-based educational classes aimed at addressing these issues. Previous research has provided empirical evidence that CBT is effective in aiding a variety of problems related to depression and anxiety. The results from the current research will be used to indicate whether the classes are an acceptable and effective intervention for low mood and stress in self-referring participants. By assessing the acceptance and feasibility of the package for this population, we will be able to gain a key insight into how similar interventions can be best delivered to adults within the community.

Participant safety and wellbeing is paramount in the study; therefore, in the participant information sheet, participants will be made aware of the fact that they should exercise normal caution in relation to their interactions with other individuals attending the classes, especially with regard to meeting outside the classes or sharing telephone numbers, email addresses and other personal information or contact details. Participants will also be told that they can seek the advice of the life skills trainer and/or the research team for advice if any problems do arise.

### Collaboration with action on depression – public engagement and knowledge exchange

AOD, the charity delivering the classes, are experienced in working with the proposed population and their facilitators will be able to assist any individuals who may need additional help during the study by giving them information about how to access alternative forms of support and the appropriate action to be taken if the participants feel their condition is deteriorating.

The self-referral, community-based approach adopted in the study may help access hard to reach individuals including young men, black and minority ethnic groups and elderly adults. However, it is acknowledged that additional consideration may be needed in the recruitment of these individuals. It would be useful for future research in this area to specifically consider minority groups both in terms of recruitment methods and also adaptation of interventions to ensure they are relevant.

#### Dissemination

Dissemination will include an executive summary, open access journal publications and conference presentations. The main journal publication will present the findings in relation to the primary and secondary research questions of the study, with a secondary health economic paper. Newsletters will be sent to all participants summarizing the study outcomes. We will also disseminate findings via the newsletters on the widely used http://www.livinglifetothefull.com website which has over 200,000 signed up members including over 9000 practitioners. AOD will also disseminate results to those receiving their newsletters and via their website. Therefore, both the professional community and individuals with depression will be made aware of the study and its findings.

#### Trial status

The project commenced in July 2012. Ethical approval has been gained and the first round of recruitment has been completed and 104 participants randomized. Four IA courses, two in Edinburgh and two in Glasgow have been delivered. The DAC participants will complete their courses in April 2013. A second recruitment phase is taking place with the aim of recruiting sufficient participants to achieve the required numbers defined by the power calculation. The expected completion date of the RCT is January 2014.

## Abbreviations

AOD: Action on Depression; cCBT: Computerized cognitive behavioral therapy; CBT: Cognitive behavioral therapy; CSQ-8: Client satisfaction questionnaire-8; CSRI: Client Service Receipt Inventory; DAC: Delayed access control; EQ5D: ; GAD7: Generalized anxiety disorder 7; HADS: Hospital Anxiety and Depression scale; HI: High intensity; IA: Immediate access; LI: Low intensity; LLTTF: Living Life to the Full; MINI: Mini International Neuropsychiatric Interview; NHS: National Health Service; NICE: National Institute for Health and Clinical Excellence; PHQ9: Patient health questionnaire-9; RCT: Randomized controlled trial; WSAS: Work and Social Adjustment Scale.

## Competing interests

CW is an author of a variety of written and cCBT resources. These are licensed through Five Areas Ltd, a company that delivers free and licensed online life skills resources based on a CBT model in a variety of settings including the NHS and voluntary and private sectors. CAM is completing a PhD with the University of Glasgow and was previously employed by both the University of Glasgow and Five Areas Ltd during the course of the development of this research protocol.

## Authors’ contributions

CW is the chief investigator of the RCT and wrote the study protocol in collaboration with CAM, JM and AM, who are co-applicants on the grant. CAM contributed to the grant application, carried out recruitment, liaised with Action on Depression regarding the delivery of the classes and will collect follow-up data. JM contributed to the writing of the protocol, and provides expert advice on key matters and wellbeing of participants. AM is the statistician for the project and provided the power calculation and analysis plan detailed in the protocol. All authors are members of the study management group and were involved in the writing of this manuscript. All authors read and approved the final manuscript.

## Authors’ information

Professor Chris Williams, Professor of Psychosocial Psychiatry, University of Glasgow. CW has experience and skills in using and evaluating self-help materials and running large randomized controlled studies, in primary care and the community.

Carrie-Anne McClay (BA Hons) Psychology, Research Assistant. CAM has recently submitted her PhD which focused on guided self-help for bulimia nervosa. CAM has an interest in new ways of delivering CBT in order to increase access to psychological therapies.

Professor Jill Morrison, Professor of General Practice, University of Glasgow. JM is an academic GP with an interest in primary care management of depression and is experienced in running randomized controlled trials in community based settings.

Dr Alex McConnachie, Assistant Director of Biostatistics, Robertson Centre for Biostatistics, University of Glasgow. AM is experienced in the design, analysis and reporting of randomized studies.
